# Bidirectional association between physical activity and muscular strength in older adults: Results from the UK Biobank study

**DOI:** 10.1093/ije/dyw054

**Published:** 2016-05-20

**Authors:** AJM Cooper, MJE Lamb, SJ Sharp, RK Simmons, SJ Griffin

**Affiliations:** 1 MRC Epidemiology Unit, Institute of Metabolic Science; 2 Primary Care Unit, Institute of Public Health, University of Cambridge, Cambridge, UK

**Keywords:** Physical activity, MVPA, muscle strength, older adults, longitudinal, bidirectional, UK Biobank

## Abstract

**Background:** The relationship between physical activity and muscular strength has not been examined in detail among older adults. The objective of this study was to examine the associations between physical activity and hand grip strength among adults aged ≥ 60 years.

**Methods:** Using data from the UK Biobank study, we included 66 582 men and women with complete baseline data and 6599 with 4.5 years of follow-up data. We used multiple linear regression models to examine the cross-sectional, longitudinal and bidirectional associations between moderate-to-vigorous physical activity (MVPA) and grip strength, adjusting for potential confounding by age, sex, height, weight, health status, education level, smoking status, Townsend deprivation index and retirement status.

**Results:** In cross-sectional analyses, grip strength and MVPA were linearly and positively associated with each other. Longitudinally, baseline MVPA was not associated with grip strength at follow-up {difference between quintile [Q] 5 and Q1 = 0.40 [95% confidence interval (CI): -0.14, 0.94]kg}, whereas baseline grip strength was associated with MVPA at follow-up [Q5 vs Q1 = 7.15 (1.18, 13.12) min/day]. People who maintained/increased time spent in MVPA did not experience any benefit in grip strength [0.08 (−0.20, 0.37) kg] whereas those who increased their grip strength spent 3.69 (0.20, 7.17) min/day extra in MVPA.

**Conclusion:** Promotion of strength-training activities may enable and maintain participation in regular physical activity among older adults.

## Introduction

Physical activity and muscular strength gradually decline after midlife.^1^^,^^2^ Only 8.5% of adults aged 60 to 69 years are physically active, for example achieve ≥ 150 min per week of moderate-to-vigorous physical activity (MVPA).^3^ Reduced muscular strength, commonly measured using grip strength,^4^ has been shown to be a strong predictor of physical performance,^5^^,^^6^ falls,^7^ disability, health-related quality of life,^8^ length of stay in hospital^9^ and mortality.^10^ Development of effective lifestyle interventions aimed at increasing participation in physical activity and improving muscular strength in older adults would benefit from a better understanding of this complex and synergistic relationship.

Several observational studies have investigated the association between physical activity and muscular strength.^11–16^ In general, findings from these studies suggest a positive association, which is consistent with results from intervention studies demonstrating that a structured exercise programme can lead to improvements in muscular strength in older adults.^17–19^ However, many of these studies have important methodological limitations. First, the observational studies have tended to be cross-sectional, thereby preventing examination of any temporal sequence. Second, the few longitudinal studies which have examined this association have mostly assessed physical activity at only one time point, thereby assuming that physical activity remains constant over time. Finally, to the best of our knowledge no studies have examined the hypothesis that the association between physical activity and muscular strength may be bidirectional—that is, an individual’s muscular strength might be an important predictor of the ability to undertake physical activity. This hypothesis is important, as evidence of a bidirectional association would suggest that lifestyle interventions may benefit from the inclusion of both adequate physical activity to improve muscular strength but also specific strength training activities to enable participation in regular physical activity.

In this study, we used repeat measures of physical activity and muscle strength during 4.5 years of follow-up in a large longitudinal UK cohort of older adults, to investigate the association between physical activity and muscle strength.

## Methods

The UK Biobank study, a large longitudinal national population-based study, was set up to investigate the role of genetic, environmental and lifestyle factors in the aetiology of diseases in mid-to-late age. The rationale and design of UK Biobank have been described elsewhere.^20^ In brief, recruitment for UK Biobank was via NHS population-based registers of people aged 40 to 69 years, living within a reasonable travelling distance of one of 22 assessment centres across England, Wales and Scotland. Recruitment invitations were mailed to 9 million people, and 502 656 UK adults (229 182 men and 273 474 women) attended for baseline measurement during 2006–10 (response rate of 5.6%).

Baseline visits took approximately 90 min and included a self-completed touch-screen questionnaire, brief computer-assisted interview, physical and functional measures and collection of blood samples. A follow-up assessment collecting the same measures was carried out in approximately 20 000 participants between 2012 and 2013. Participants were invited to attend follow-up assessment via email or letter, with an overall response rate of 21%.

Participants provided full informed consent to participate in UK Biobank. This study was covered by the generic ethical approval for UK Biobank studies from the NHS National Research Ethics Service (Ref: 11/NW/0382).

### Measures

#### Physical activity

Physical activity was assessed using an adaptation of the self-report International Physical Activity Questionnaire (IPAQ) short form at baseline and follow-up.^21^ Participants were asked questions such as how many days in a typical week they spent in moderate-intensity physical activity (e.g. carrying light loads and cycling) and in vigorous-intensity physical activity (e.g. fast cycling, aerobics and heavy lifting). For each of the categories of physical activity engaged in at least once per week, participants were then asked to provide information on the duration spent in that activity on a typical day. To derive time spent in MVPA, the activity frequency was multiplied by the duration spent in the activity.

#### Grip strength

We used grip strength as a surrogate measure of overall muscle strength, as it has been shown to be strongly related to lower extremity muscle power, knee extension torque and calf cross-sectional area.^22^ It is also the measure for assessing general muscular strength recommended by the European Working Group on Sarcopenia in Older People (EWGSOP).^5^ Maximal grip strength was measured using a hydraulic hand dynamometer (Jamar J00105) at baseline and follow-up. Participants were asked to sit upright in a chair with their forearms placed on armrests and elbows placed against their sides at a 90° angle. Participants were instructed to squeeze the handle of the dynamometer as strongly as they could for 3s while keeping their wrist straight.^23^ Grip strength was measured in both hands and the highest value was used for these analyses.

#### Covariates

Covariate data were collected at baseline. Socio-demographic factors included age, sex and education level. Education level was categorized as having: (i) a college or university degree, (ii) A levels/AS levels or equivalent, (iii) O levels/GCSEs or equivalent, (iv) CSEs or equivalent, (v) NVQ or HND or HNC or equivalent or (v) other qualification. Retirement from main occupation was coded as yes/no. Smoking status was self-reported and coded as (i) current smoker, (ii) former smoker or (iii) never smoker. Townsend deprivation index scores were calculated based on participants’ home postal codes. Height was measured without shoes with participants standing with their back against a vertical scale (SECA 240-cm height measure). Weight was measured without shoes (Tanita BC418MA or standard scales). Overall health status was self-reported at baseline and follow-up and categorized as: (i) excellent, (ii) good, (ii) fair or (iv) poor.

### Statistical analysis

Participant characteristics for those included in the cross-sectional and longitudinal analyses were summarized using means [standard deviation (SD)] or frequencies (%). To allow for possible non-linear associations, we categorized exposure data into quintiles. We estimated associations between MVPA and grip strength by fitting multiple linear regression models and calculating adjusted means and 95% confidence intervals (95% CIs) of the outcome variable (continuous) at each level of the exposure variable, setting all other covariates in the model to their mean values in the sample. In Model 1, we adjusted for age, sex, height and weight. Model 2 was additionally adjusted for health status and education level. In preliminary analyses, Model 2 was also adjusted for smoking status, Townsend deprivation index and retirement status, but these variables were not associated with MVPA and muscular strength, and as their inclusion in the models did not influence the direction or magnitude of associations (< 10% change in magnitude), they were not included in the final model. All data were analysed using STATA software version 13.1.

#### Association of MVPA with grip strength

We examined the cross-sectional association between MVPA and grip strength at baseline by calculating adjusted means of grip strength within quintiles of MVPA. In a longitudinal analysis, we examined the association between MVPA at baseline and grip strength at follow-up. Mean values for grip strength were calculated according to quintiles of MVPA. Finally, we examined the association between change in MVPA from baseline to follow-up and change in grip strength, adjusted for baseline measures of MVPA and grip strength. For these analyses, mean changes in grip strength were calculated according to both (i) participants who maintained/increased their MVPA between baseline and follow-up vs those who had decreased over follow-up, and (ii) quintiles of change in MVPA.

#### Association of grip strength with MVPA

We performed the same analyses as above, but this time using grip strength as the exposure variable and MVPA as the outcome variable. For the association between change in grip strength and change in MVPA, mean changes in MVPA were calculated according to both (i) participants who maintained/increased their grip strength from baseline to follow-up compared with those who had a reduction in grip strength, and (ii) quintiles of change in grip strength.

To examine for linear trends across increasing quintiles of the exposure variables, we included exposure quintile as a continuous variable in the model, and used Wald tests of the null hypothesis that the true value of the parameter associated with this variable was zero. We included multiplicative interactions in the model to explore whether the associations between MVPA and grip strength, and vice versa, were modified by age (< 65 vs ≥  65 years) and sex. Associations between baseline and follow-up measures of MVPA and grip strength were estimated using Pearson correlation coefficients.

## Results

Among the 502 656 participants who attended for the UK Biobank baseline examination, 66 582 participants were aged ≥ 60 years and had complete data on all variables at baseline, so were included in the cross-sectional analyses. Of the approximate 20 000 participants who attended for a follow-up assessment, 6599 were aged ≥ 60 years at baseline and had complete data on all variables. As shown in Table 1, the mean (SD) age of the cross-sectional sample was 66.6 (1.8) years, with a similar percentage of men and women. Over 75% of participants at baseline reported having good or excellent health; 35% had been educated to college or degree level and over 80% reported being retired from their main occupation (Table 1). Between baseline and follow-up, the mean (SD) decrease in grip strength was 7.0 (6.5) kg (Table 2).

**Table 1.** Characteristics of participants in the cross-sectional sample

	Men (*n* = 33403)	Women (*n* = 33179)	Men & women combined (*n* = 66 582)
Age, years, mean (SD)	66.6 (1.8)	66.6 (1.8)	66.6 (1.8)
Height, cm, mean (SD)	174.8 (6.5)	161.5 (6.0)	168.2 (9.1)
Weight, kg, mean (SD)	83.9 (13.0)	70.3 (12.6)	77.1 (14.5)
Health status			
Excellent	5522 (16.5)	5439 (16.4)	10 961 (16.5)
Good	20081 (60.1)	21174 (63.8)	41 255 (62.0)
Fair	6676 (20.0)	5766 (17.4)	12 442 (18.7)
Poor	1124 (3.4)	800 (2.4)	1924 (2.9)
Education level			
College or university degree	12 802 (38.3)	10 523 (31.7)	23 325 (35.0)
A levels/AS levels or equivalent	3768 (11.3)	4269 (12.9)	8037 (12.1)
O levels/GCSEs or equivalent	8161 (24.4)	11 598 (35.0)	19 759 (29.7)
CSEs or equivalent	718 (2.2)	919 (2.8)	1637 (2.5)
NVQ or HND or HNC or equivalent	4939 (14.8)	1664 (5.0)	6603 (9.9)
Other	3015 (9.0)	4206 (12.7)	7221 (10.9)
Retired from main occupation	26 619 (80.0)	28 182 (85.4)	54 801 (82.7)
Time spent in MVPA, min/day, median (interquartile range)	28.6 (8.6, 64.3)	32.1 (10.0, 68.6)	30.0 (8.6, 65.7)
Grip strength, kg, mean (SD)	38.8 (8.0)	22.8 (5.8)	30.8 (10.6)

Results are *n* (%) unless stated otherwise.

**Table 2.** Characteristics of participants in the prospective cohort^a^

	**Baseline**	**Follow-up**	**Change**
Age, years, mean (SD)	63.8 (2.5)	68.1 (2.4)	4.3 (0.9)
Weight, kg, mean (SD)	77.2(14.5)	76.6 (14.5)	−0.6 (4.3)
Health status			
Excellent	1383 (21.0)	1036 (15.7)	−347 (−5.3)
Good	4143 (62.8)	4208 (63.8)	65 (1.0)
Fair	959 (14.5)	1203 (18.2)	244 (3.7)
Poor	114 (1.7)	152 (2.3)	38 (0.6)
Time spent in MVPA, min/day, median (interquartile range)	27.1 (8.6, 60.0)	27.9 (10.0, 60.0)	0 (−20.0, 17.1)
Grip strength, kg, mean (SD)	33.5 (10.7)	26.5 (10.1)	−7.0 (6.5)

Results are *n* (%) unless stated otherwise.

a *n* = 6599 (*n* = 3539 men and *n* = 3060 women).

### 

#### Association of MVPA with grip strength

**Figure 1.**
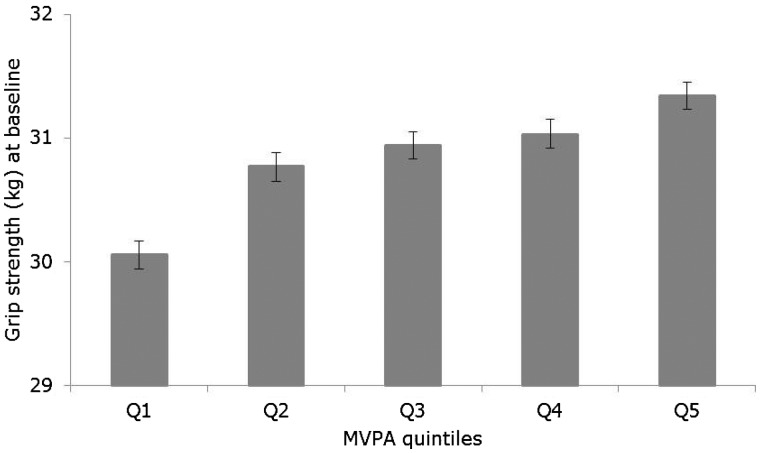
Cross-sectional association between moderate-to-vigorous physical activity (MVPA) and grip strength (total *n*=66 582; *n* for Q1=13 325, Q2=13 515, Q3=13 314, Q4=13 118, Q5=13 310). Values are means and 95% confidence intervals estimated from a linear regression model with grip strength at baseline as the outcome, MVPA quintiles at baseline as the exposure, and adjusted for age, sex, height, weight, health status and education level.

At baseline, men and women who reported doing more MVPA had higher values for grip strength, and this association was linear across increasing quintiles of MVPA (*P* for linear trend < 0.001) (Figure 1). On average, those in the highest quintile of MVPA had a grip strength which was 1.28 (95% CI: 1.08, 1.48) kg greater than those in the lowest quintile, after adjusting for known potential confounders (Supplementary Table 1, available as Supplementary data at *IJE* online).

**Figure 2.**
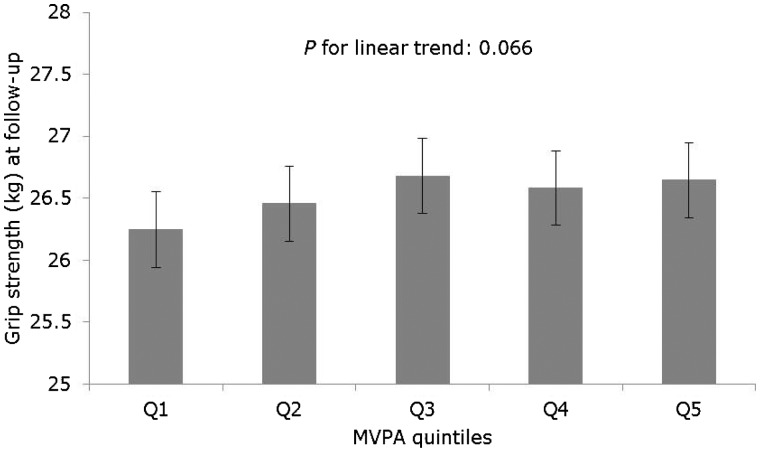
Prospective association between baseline moderate-to-vigorous physical activity (MVPA) and grip strength at 4.5 year follow-up (*n*=6599; *n* for Q1=1330, Q2=1320, Q3=1347, Q4=1297, Q = 1305). Values are means and 95% confidence intervals estimated from a linear regression model with grip strength at follow-up as the outcome, MVPA quintiles at baseline as the exposure, and adjusted for age, height, weight, health status, education level and baseline grip strength.

**Figure 3.**
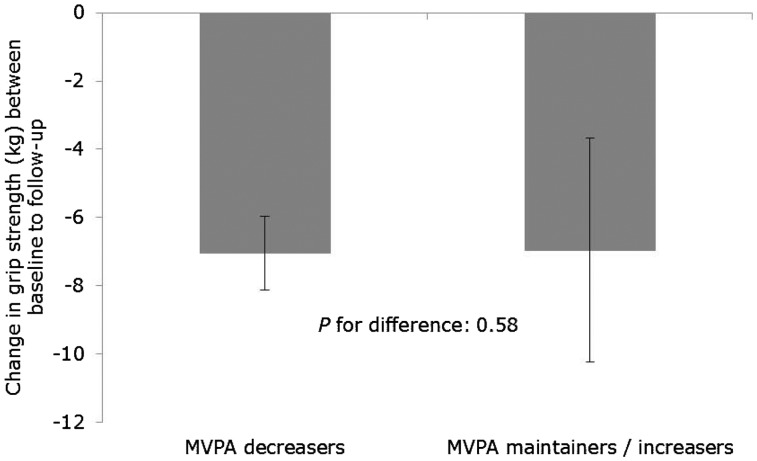
Association between change in moderate-to-vigorous physical activity (MVPA) and change in grip strength between baseline and 4.5 year follow-up (total *n*=6599, decreasers *n*=3538; maintainers / increasers *n*=3061). Values are means and 95% confidence intervals estimated from a linear regression model with change in grip strength as the outcome, MVPA decreasers versus maintainers / increasers as the exposure, and adjusted for age, sex, height, weight, baseline- and follow-up health status, education level and baseline MVPA and grip strength at baseline.

Figure 2 shows the association between quintiles of baseline MVPA and mean grip strength at follow-up. Although the association between baseline MVPA and follow-up grip strength was positive, the linear trend across quintiles was not significant (*P* for linear trend: 0.066) and there was little difference in grip strength comparing the highest with lowest quintiles of MVPA (0.40; 95% CI: –0.14, 0.94 kg) (Supplementary Table 2, available as Supplementary data at *IJE* online). There was no evidence to suggest that change in grip strength was different in those who reported maintaining/increasing the amount of time spent in MVPA compared with those whose time in MVPA decreased over follow-up [difference: 0.08 (–0.20, 0.37) kg; *P* for difference: 0.58) (Figure 3; Supplementary Table 3, available as Supplementary data at *IJE* online), a finding which was consistent with the analysis examining this association across increasing quintiles of MVPA (Supplementary Figure 1, available as Supplementary data at *IJE* online).

#### Association of grip strength with MVPA

**Figure 4.**
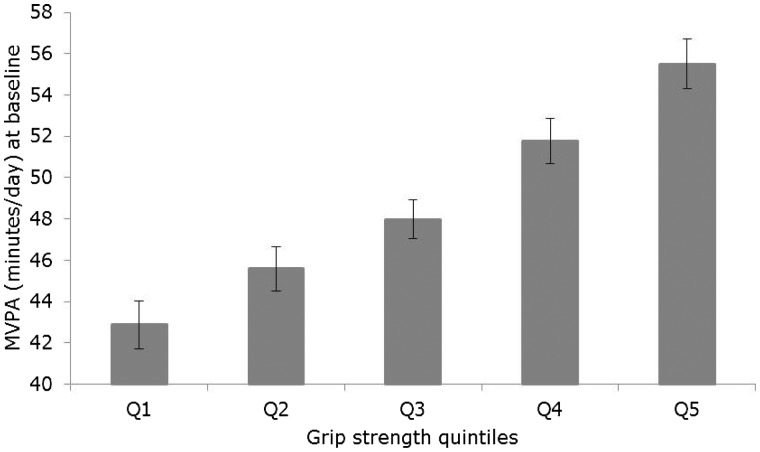
Cross-sectional association between grip strength and moderate-to-vigorous physical activity (MVPA) (*n*=66 582; *n* for Q1=13 429, Q2=13 560, Q3=13 170, Q4=13 535, Q5=12 888). Values are means and 95% confidence intervals estimated from a linear regression model with time spent in MVPA at baseline as the outcome, grip strength quintiles at baseline as the exposure, and adjusted for age, sex, height, weight, health status and education level.

There was a positive linear association between quintiles of baseline grip strength and time spent in MVPA at baseline (*P* for linear trend < 0.001) (Figure 4). On average, those in the highest quintile of grip strength spent 12.63 (95% CI: 10.22, 15.05) min more time in MVPA per day than those in the lowest grip strength quintile (Supplementary Table 1).

**Figure 5.**
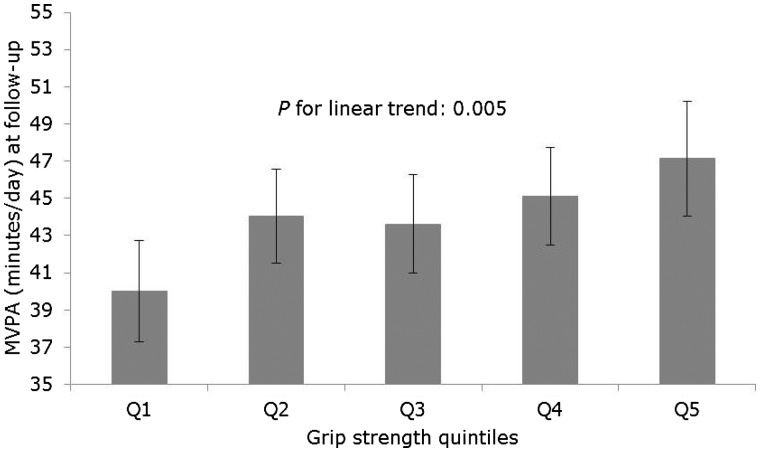
Prospective association between baseline grip strength and moderate-to-vigorous physical activity (MVPA) at 4.5 year follow-up (*n*=6599; *n* for Q1=1627, Q2=1382, Q3=1007, Q4=1502, Q5=1081). Values are means and 95% confidence intervals estimated from a linear regression model with MVPA at follow-up as the outcome, grip strength quintiles at baseline as the exposure, and adjusted for age, height, weight, health status, education level and baseline MVPA.

**Figure 6.**
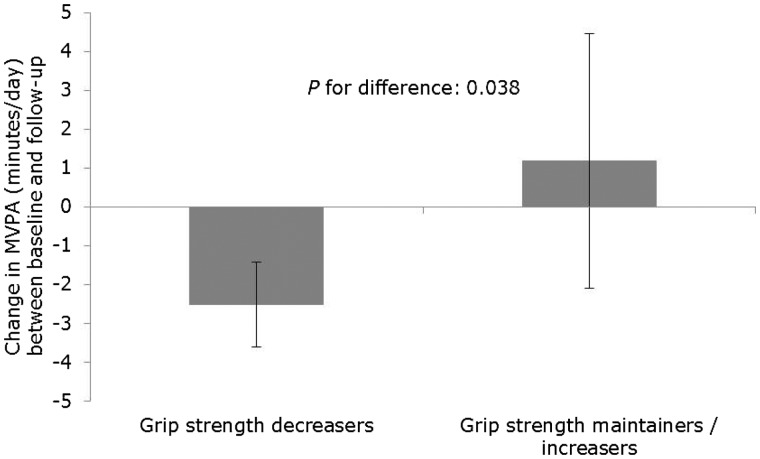
Association between change in grip strength and change in moderate-to-vigorous physical activity (MVPA) over 4.5 years of follow-up (total *n*=6599, decreasers *n*=5898; maintainers / increasers *n*=701). Values are means and 95% confidence intervals estimated from a linear regression model with change in MVPA as outcome, grip strength decreasers versus maintainers / increasers as the exposure, and adjusted for age, sex, height, weight, baseline- and follow-up health status, education level and baseline grip strength and MVPA.

The association between quintiles of baseline grip strength and MVPA at follow-up is shown in Figure 5. MVPA at follow-up was higher across increasing quintiles of baseline grip strength (*P* for linear trend: 0.005), such that those in the highest quintile of grip strength spent 7.15 (95% CI: 1.18, 13.12) min/day more time in MVPA at follow-up than those in the lowest grip strength quintile (Supplementary Table 2). In adjusted analyses, participants who maintained/increased their grip strength between baseline and follow-up spent 3.69 (95% CI: 0.20, 7.17) min/day more time in MVPA compared with those who experienced a decrease in grip strength at follow-up (*P* for difference: 0.038) (Figure 6; Supplementary Table 3), for which the relationship appeared to be linear across increasing quintiles of grip strength (Supplementary Figure 2).

#### Interaction

There was no evidence that the associations between MVPA and grip strength, and vice versa, differed by age group or sex (all *P-*values for interaction > 0.05). MVPA at baseline was moderately correlated with MVPA at follow-up (*r* = 0.47; *P* < 0.001) and baseline grip strength was strongly correlated with grip strength at follow-up (*r* = 0.81; *P* < 0.001).

## Discussion

We examined the cross-sectional, longitudinal and bidirectional associations between physical activity and muscular strength in a large cohort of men and women in early old age. Findings from the cross-sectional analyses showed that muscle strength and MVPA were positively associated with each other. In the longitudinal analyses, we demonstrated that baseline grip strength was linearly and positively associated with MVPA at follow-up, whereas MVPAat baseline was only weakly associated with grip strength at follow-up. By taking advantage of the availability of repeated measures of the exposure and outcome in bidirectional analyses, we have been able to show that older adults who maintained/improved their muscle strength were more likely to increase their levels of physical activity over follow-up, whereas those who increased their level of physical activity did not increase their muscular strength.

Our cross-sectional findings are consistent with most previous observational studies which have demonstrated a positive association between physical activity and muscle strength in older adults.^12–16^ Our finding that baseline physical activity was associated with muscle strength at follow-up is consistent with the findings of Cooper *et al*. who showed, using data from the National Survey of Health and Development study, that although self-reported physical activity was associated with grip strength in a cross-sectional analysis, physical activity at 36 and 43 years was not associated with grip strength at 53 years.^24^

Compared with previous studies,^11–16^ our study is novel because we were able to use measures of physical activity and muscle strength at two different time points to examine the possibility that the relationship between physical activity and strength might be bidirectional among older adults. Using both baseline and follow-up data in these analyses, we show that despite physical activity and grip strength being inter-related, it appears that those who maintain/improve their grip strength are more likely to increase the amount of time they spend being physically active, whereas an increase in MVPA does not appear to lessen the decline in grip strength. What is more, our finding that the relationship between change in grip strength and MVPA is linear suggests that minimizing loss of strength, and not necessarily increasing/maintaining strength, will still be related to a less rapid decline in MVPA.

An important factor to consider when interpreting results from bidirectional analyses is the difference in random measurement error associated with the exposure and outcome measures. IPAQ has previously been shown to be valid for assessing physical activity in older adults; the correlation between IPAQ and objectively measured physical activity by accelerometry is *ρ* = 0.37 for men and *ρ* = 0.43 for women (*P* < 0.01), indicating a moderate correlation.^25^ In contrast, grip strength is measured very precisely.^4^ The bias introduced by marked differences in measurement error depends on whether the variable measured with the least precision is analysed as the exposure or outcome variable. When the imprecise measure is analysed as the exposure variable, it acts to bias the effect estimate towards the null. In contrast, when the imprecise measure is analysed as the outcome variable, the magnitude of effect is estimated accurately, but the standard error of the estimate is increased and the corresponding confidence intervals widened, making the result less likely to be significant.^26^ Consequently, under the assumption that the associations between physical activity and grip strength are bidirectionally equivalent, as grip strength is measured with greater precision it will always appear that it is the stronger predictor of physical activity rather than vice versa. Although a direct comparison of regression estimates is therefore difficult, our findings do suggest that an individual’s muscle strength does play an important role in enabling participation in physical activity. Future studies with objective measures of physical activity, in different age groups, are needed to confirm our findings and to establish whether there is a point in life when muscular strength becomes increasingly important as a cause rather than a consequence of physical activity.

There are several plausible explanations for our findings. First, resistance training has been shown to be associated with an increase in physical activity in an intervention study of older adults aged 61 to 77 years,^27^ which is intuitive since a certain amount of muscular strength is required to undertake physical activity with ease. Second, our longitudinal analyses included 4.5 years of follow-up time and the IPAQ questionnaire only asked about physical activity in a typical week. Previous findings suggest that physical activity over the life course might have a stronger relationship with muscle strength.^24^ Finally, the activities undertaken by UK Biobank participants might not be of the correct type, intensity or frequency to improve upper body strength.

Our study has a number of important strengths, including the large sample size, objective measures of muscle strength and long-term follow-up. Also, whereas assessment of physical activity by self-report generally leads to an overestimate of physical activity levels, self-report questionnaires have been shown to be sensitive to changes in physical activity,^28^^,^^29^ as suggested by the moderate correlation we found between MVPA measured at baseline and follow-up. Our study also has several limitations. First, UK Biobank participants are healthier than the general UK older adult population, thereby reducing the generalizability of our findings.^30^ Second, even though the physical activity questionnaire used in UK Biobank has been shown to be valid for grouping individuals according to their level of physical activity,^31^ we cannot exclude the possibility that bias will have affected our findings due to misreporting. Indeed, it has previously been shown that 59.7% of adults aged 60 to 69 years report meeting the physical activity guidelines of ≥ 150 min/week of MVPA, yet only 8.5% actually met these guidelines when activity was measured objectively.^3^ Third, grip strength provides a simple and inexpensive measure of general muscle strength, but it may not be a good surrogate for lower limb strength.^32^ Finally, although we were able to adjust for important confounders, we cannot exclude the possibility of confounding by unmeasured factors or residual confounding by factors imprecisely measured.

Our findings suggest that to reduce the burden of disability, dependency, morbidity and premature mortality in older adults, the interdependence of physical activity and muscle strength should be considered. Interventions aimed at promoting physical activity might incorporate muscle-strengthening exercises to enhance their effectiveness and to gain the independent benefits of increased muscle strength.

## Supplementary Data

Supplementary data are available at *IJE* online.

## Funding

This work was supported by the Medical Research Council [programme number: MC_UU_12015/4] and has been conducted using the UK Biobank Resource.

**Conflict of interest**: The authors declare that there is no conflict of interests regarding the publication of this paper.

##

Key Messages

- The relationship between physical activity and muscular strength has not been examined in detail among older adults.
- This study found that people who maintained/increased their time spent being physically active did not experience any benefit in muscular strength, whereas those who increased their strength did spend more time being physically active.
- Promotion of activities aimed at improving/maintaining muscular strength might enable older adults to remain physically active.

## Supplementary Material

Supplementary Data
