# Supplementary material for: Bidirectional association between physical activity and muscular strength in older adults: Results from the UK Biobank study
Source: Int J Epidemiol. 2016 May 20;46(1):141–8. doi: 10.1093/ije/dyw054 (PMC5407153; doi:10.1093/ije/dyw054)
Supplement: Supplementary Data [file dyw054_supp.pdf]

Supplementary Table 1. Cross sectional associations between moderate-to-vigorous physical activity (minutes/day) and hand grip strength (kg) (*n*=66 582)

	**Quintiles of baseline MVPA & continuous grip strength at baseline (kg)†**					***P* for linear trend**	**Difference (95% CI) between Q5 and Q1**
	**Q1**	**Q2**	**Q3**	**Q4**	**Q5**		
**Model 1**	29.78 (29.67, 29.89)	30.74 (30.63, 30.86)	31.00 (30.89, 31.11)	31.13 (31.02, 31.24)	31.48 (31.37, 31.60)	<0.001	1.70 (1.50, 1.90)
**Model 2**	30.06 (29.94, 30.17)	30.77 (30.65, 30.88)	30.94 (30.83, 31.05)	31.03 (30.92, 31.15)	31.34 (31.23, 31.45)	<0.001	1.28 (1.08, 1.48)
	**Quintiles of baseline grip strength & continuous MVPA at baseline (minutes/day)††**						
	**Q1**	**Q2**	**Q3**	**Q4**	**Q5**		
**Model 1**	41.02 (39.87, 42.16)	44.89 (43.82, 45.96)	47.92 (46.99, 48.85)	52.62 (51.53, 53.71)	57.34 (56.14, 58.53)	<0.001	16.32 (13.92, 18.72)
**Model 2**	42.87 (41.73, 44.01)	45.59 (44.53, 46.66)	47.98 (47.05, 48.91)	51.76 (50.68, 52.85)	55.50 (54.31, 56.70)	<0.001	12.63 (10.22, 15.05)

Model 1 adjusted for: age, sex, height and weight.
Model 2 adjusted for: Model 1 covariates plus baseline health status and education level.
† Values for baseline grip strength are in kg.
†† Values for baseline MVPA are in minutes per day.

Supplementary Table 2. Prospective associations between moderate-to-vigorous physical activity (minutes/day) and hand grip strength (kg) (*n*=6599)

	**Quintiles of baseline MVPA and continuous grip strength at follow-up (kg)*†**					***P* for linear trend**	**Difference (95% CI) between Q5 and Q1**
	**Q1**	**Q2**	**Q3**	**Q4**	**Q5**		
**Model 1**	26.12 (25.82, 26.42)	26.47 (26.16, 26.77)	26.71 (26.41, 27.01)	26.62 (26.31, 26.92)	26.70 (26.40, 27.01)	0.008	0.58 (0.05, 1.12)
**Model 2**	26.25 (25.94, 26.55)	26.46 (26.15, 26.76)	26.68 (26.38, 26.98)	26.58 (26.28, 26.88)	26.65 (26.34, 26.95)	0.066	0.40 (-0.14, 0.94)
	**Quintiles of baseline grip strength and continuous MVPA at follow-up (minutes/day)**††**						
	**Q1**	**Q2**	**Q3**	**Q4**	**Q5**		
**Model 1**	39.42 (36.72, 42.13)	43.78 (41.23, 46.32)	43.64 (40.97, 46.30)	45.50 (42.90, 48.11)	47.79 (44.71, 50.86)	<0.001	8.36 (2.42, 14.31)
**Model 2**	40.00 (37.29, 42.71)	44.04 (41.50, 46.58)	43.62 (40.96, 46.27)	45.11 (42.51, 47.72)	47.14 (44.06, 50.22)	0.005	7.15 (1.18, 13.12)

*Model 1 adjusted for: baseline grip strength, age, sex, height and weight.
*Model 2 adjusted for: Model 1 covariates plus baseline health status and education level.
† Values for change in grip strength between baseline and follow-up are in kg.
**Model 1 adjusted for: baseline MVPA, age, sex, height and weight.
**Model 2 adjusted for: Model 1 covariates plus baseline health status and education level.
†† Values for change in MVPA between baseline and follow-up are in minutes per day.

Supplementary Table 3. Associations between change in moderate-to-vigorous physical activity (minutes/day) and change in hand grip strength (kg) (*n*=6599)

	**Change in grip strength (kg) by change in MVPA category†**		**Difference (95% CI) between maintainers /increasers vs. decreasers**	***P* for difference**
	**Decreasers (*n*=3,538)**	**maintainers / increasers (*n*=3,061)**		
**Model 1**	-7.10 (-7.29, -6.91)	-6.90 (-7.10, -6.69)	0.20 (-0.08, 0.49)	0.17
**Model 2**	-7.04 (-7.23, -6.86)	-6.96 (-7.17, -6.76)	0.08 (-0.20, 0.37)	0.58
	**Change in MVPA (minutes/day) by change in grip strength category††**			
	**Decreasers (*n*=5,898)**	**maintainers / increasers (*n*=701)**		
**Model 1**	-2.57 (-3.67, -1.48)	1.69 (-1.60, 4.97)	4.26 (0.77, 7.75)	0.017
**Model 2**	-2.51 (-3.60, -1.43)	1.17 (-2.10, 4.45)	3.69 (0.20, 7.17)	0.038

Model 1 adjusted for: baseline MVPA, baseline grip strength, age, sex height and weight.
Model 2 adjusted for: Model 1 covariates plus baseline and follow-up health status and education level.
† Values for change in grip strength between baseline and follow-up are in kg
†† Values for change in MVPA between baseline and follow-up are in minutes per day

Supplementary Figure 1

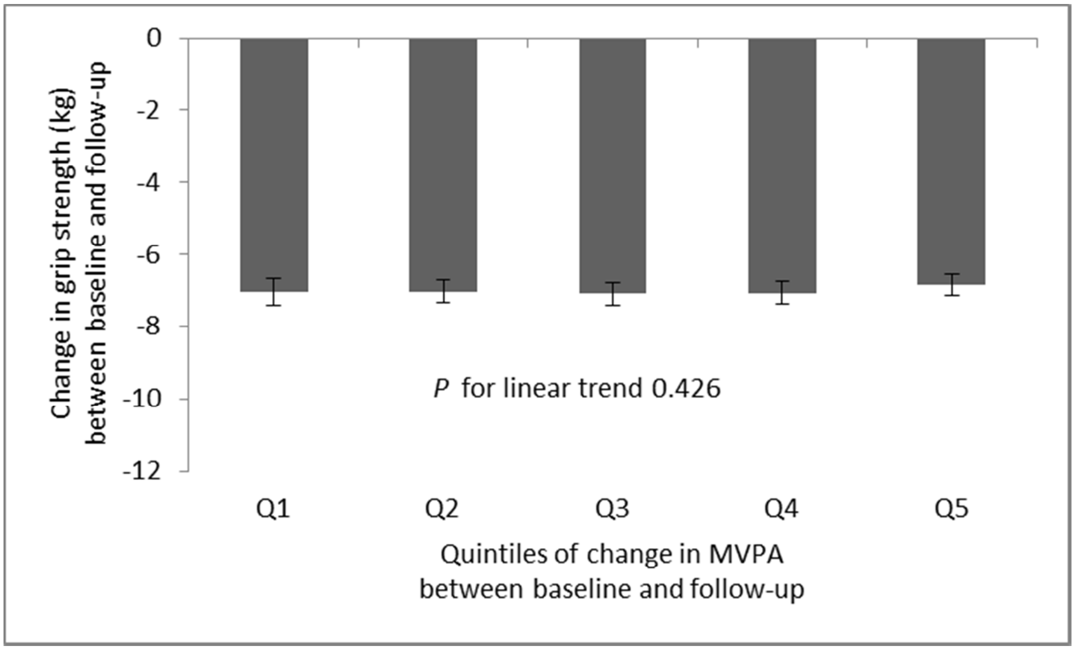

Sup. Fig. 1 Association between change in moderate-to-vigorous physical activity (MVPA) and change in grip strength between baseline and 4.5 year follow-up (total *n*=6599, Q1 *n*=1332; Q2 *n*=1308; Q3 *n*=1320; Q4 *n*=1321; Q5 *n*=1318). Values are means and 95% confidence intervals estimated from a linear regression model with change in grip strength as the outcome, MVPA decreasers versus maintainers / increasers as the exposure, and adjusted for age, sex, height, weight, baseline- and follow-up health status, education level and baseline MVPA and grip strength at baseline.

Supplementary Figure 2

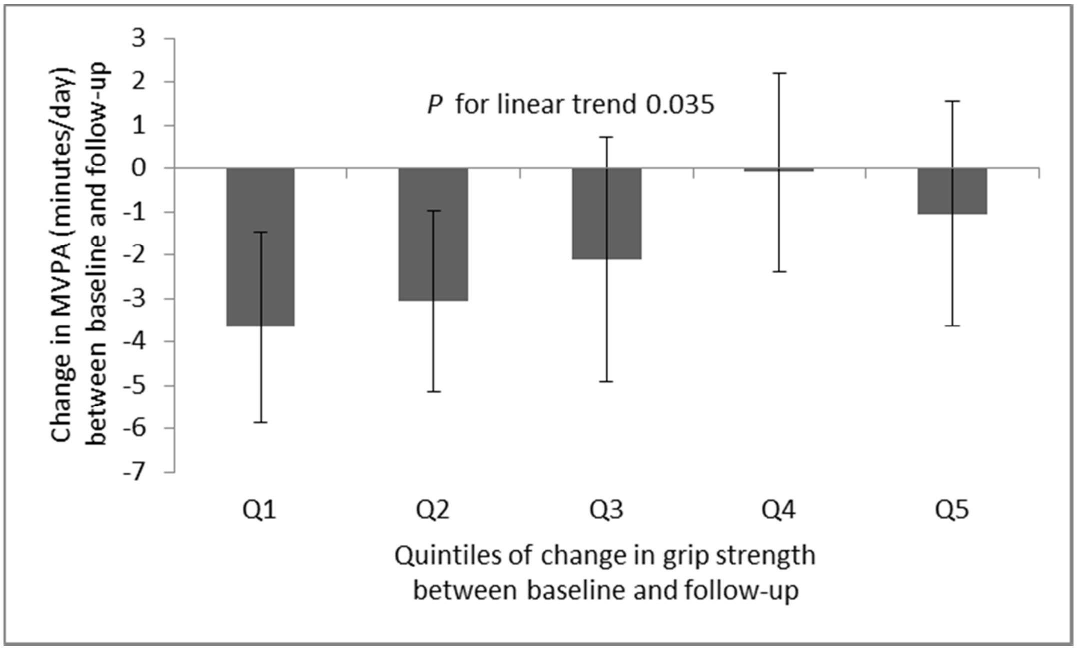

Sup. Fig. 2 Association between change in grip strength and change in moderate-to-vigorous physical activity (MVPA) over 4.5 years of follow-up (total *n*=6599, Q1 *n*=1628; Q2 *n*=1597; Q3 *n*=869; Q4 *n*=1348; Q5 *n*=1157). Values are means and 95% confidence intervals estimated from a linear regression model with change in MVPA as outcome, grip strength decreasers versus maintainers / increasers as the exposure, and adjusted for age, sex, height, weight, baseline- and follow-up health status, education level and baseline grip strength and MVPA.
